# Eficácia da Hidratação Oral na Prevenção da Nefropatia Induzida por Contraste em Indivíduos Submetidos a Intervenções Coronárias Eletivas

**DOI:** 10.36660/abc.20220529

**Published:** 2023-02-13

**Authors:** Mariana Rodrigues Pioli, Renata Muller Couto, José de Arimatéia Francisco, Diego Quilles Antoniassi, Célia Regina de Souza, Matheus Ynada de Olivio, Gabriel Forato Anhê, Silvio Giopatto, Andrei C. Sposito, Wilson Nadruz, Otavio Rizzi Coelho-Filho, Rodrigo Modolo

**Affiliations:** 1 Universidade Estadual de Campinas Faculdade de Ciências Médicas Departamento de Medicina Translacional Campinas SP Brasil Universidade Estadual de Campinas Faculdade de Ciências Médicas – Departamento de Medicina Translacional, Programa de Farmacologia, Campinas, SP – Brasil; 2 Universidade Estadual de Campinas Faculdade de Ciências Médicas Departamento de Medicina Interna Campinas SP Brasil Universidade Estadual de Campinas Faculdade de Ciências Médicas – Departamento de Medicina Interna – Divisão de Cardiologia, Campinas, SP – Brasil; 3 Universidade Estadual de Campinas Laboratório de Aterosclerose e Biologia Vascular Campinas SP Brasil Universidade Estadual de Campinas (UNICAMP) – Laboratório de Aterosclerose e Biologia Vascular (Atherolab), Campinas, SP – Brasil; 4 Universidade Estadual de Campinas Campinas SP Brasil Universidade Estadual de Campinas (UNICAMP) – Disciplina de Cardiologia, Campinas, SP – Brasil

**Keywords:** Injúria Renal Aguda, Meios de Contraste, Angioplastia, Cateterismo Cardíaco

## Abstract

**Fundamento:**

A nefropatia induzida por contraste (NIC) é definida como deterioração da função renal, representada por um aumento da creatinina sérica ≥25% ou ≥0,5 mg/dL até 72 horas após a exposição ao meio de contraste iodado (MCI). A medida preventiva mais eficaz até o momento é a hidratação venosa (HV). Pouco se sabe sobre a eficácia da hidratação oral (HO) ambulatorial.

**Objetivo:**

Investigar se a HO ambulatorial com água é tão eficaz quanto a HV com solução salina a 0,9% na prevenção de NIC em procedimentos coronarianos eletivos.

**Métodos:**

Neste estudo observacional retrospectivo, foram analisados prontuários médicos e dados laboratoriais para coletar dados de indivíduos submetidos a procedimentos coronarianos percutâneos com MCI. Os dados coletados entre 2012 e 2015 avaliaram indivíduos que foram submetidos à HV e entre 2016 e 2020 (após a implementação de um protocolo de HO), os indivíduos que foram submetidos à HO em casa antes e depois de procedimentos coronarianos, conforme orientação da equipe de enfermagem. A significância estatística adotada foi de α=0,05.

**Resultados:**

No total, 116 pacientes foram incluídos neste estudo, 58 no grupo HV e 58 no grupo HO. Observou-se incidência de NIC de 15% (9/58) no grupo que recebeu HV e 12% (7/58) no grupo que recebeu HO (p=0,68).

**Conclusão:**

O protocolo de HO realizado pelo paciente parece ser tão eficaz quanto o protocolo de HV hospitalar na proteção renal de indivíduos suscetíveis a desenvolver NIC em intervenções coronarianas eletivas. Essas descobertas devem ser testadas em ensaios mais abrangentes.

## Introdução

A nefropatia induzida por contraste (NIC) foi descrita em 1954 por Bartels et al.^1^ e definida por Mehran et al.^2^ como um aumento na creatinina sérica ≥25% ou ≥0,5mg/dL até 72 horas após a exposição a meio de contraste iodado (MCI). É considerada uma iatrogênese altamente incidente em intervenções coronarianas, atingindo até 2% da população exposta ao MCI^3 , 4^ ou até 50% em populações de alto risco.^5 , 6^ Além disso, está fortemente associada a desfechos clínicos desfavoráveis, como morbidade e mortalidade a longo prazo.^7 , 8^

O desenvolvimento da NIC está associado tanto a características do MCI^9 , 10^ como à condição clínica do paciente, já que indivíduos com insuficiência renal preexistente, diabetes e idosos são mais propensos a esse desfecho.^11 , 12^ Medidas profiláticas foram relatadas como eficazes para reduzir a incidência de NIC, a saber: a identificação de fatores de risco nos pacientes, o uso do menor volume possível de MCI e a proteção renal antes e depois do procedimento por meio de hidratação venosa (HV) com solução salina a 0,9%.^13 , 14^

Apesar de ser seguro e recomendado por várias diretrizes,^15 - 18^ a HV tem alguns aspectos que às vezes limitam sua aplicação, como o aumento do tempo de internação, gerando altos custos para o hospital e desconforto para os pacientes. Portanto, uma estratégia alternativa como a hidratação oral (HO) poderia ser uma opção importante já que, além de causar a expansão adequada do volume, é fácil de ser realizada antes e depois do procedimento, econômica e confortável para o paciente.

Estudos anteriores mostraram que a HO pode reduzir a incidência de NIC,^19^ mas outros resultados mostraram que a HV é superior.^20^ Em vista desses resultados conflitantes, pretendemos investigar se a HO com água, antes e depois da administração de MCI, é tão eficaz quanto a HV com solução salina a 0,9%, na proteção renal de indivíduos suscetíveis a desenvolver NIC em procedimentos coronarianos eletivos para cateterismo cardíaco e intervenções coronarianas.

## Métodos

Este estudo foi aprovado pelo Comitê de Ética em Pesquisa da Faculdade de Ciências Médicas da UNICAMP (#4.124.863 e CAAE: 33427720.2.0000.5404). Por tratar-se de um estudo observacional retrospectivo, os participantes da pesquisa foram dispensados do consentimento informado, aprovado pelo comitê de ética.

### População de pacientes e definição de NIC

Selecionaram-se 116 pacientes consecutivos que foram submetidos a procedimentos eletivos de cateterização cardíaca e/ou intervenção coronariana percutânea (ICP), entre janeiro de 2012 e janeiro de 2020, com alto risco de desenvolver NIC (critérios descritos abaixo). Este é um estudo de centro único do Laboratório de Cateterização Cardíaca do Hospital das Clínicas da Universidade Estadual de Campinas - UNICAMP.

Todos os pacientes foram submetidos à avaliação do histórico médico com a equipe de enfermagem para avaliar o risco de desenvolver NIC, que foi definida como um aumento da creatinina sérica ≥25% ou ≥0,5mg/dL até 72 horas após a exposição ao MCI.^2^ Os pacientes com creatinina sérica >1,5mg/dL ou taxa de filtração glomerular estimada (TFGe) entre 40-60mL/min, foram automaticamente incluídos no “Protocolo de Prevenção de Nefropatia Induzida por Contraste”.

No caso de pacientes com níveis normais de creatinina sérica, outras características clínicas, que estão relacionadas à deterioração da função renal, também foram avaliadas, a fim de classificar o paciente como grupo de risco para desenvolver NIC. Os fatores considerados para avaliação de risco foram: idosos (>75 anos), comorbidades preexistentes como *diabetes mellitus* (DM), hipertensão e doença renal crônica (DRC), instabilidade hemodinâmica e também uso de drogas nefrotóxicas.^12 , 21^ Os critérios de exclusão foram os seguintes: pacientes em diálise; casos de urgência e emergência; indivíduos com insuficiência cardíaca congestiva em classe funcional III e IV; e sem informações nos prontuários médicos.

### Protocolo de estudo

Até 2015, o paciente que foi incluído no “Protocolo de Prevenção de NIC” deveria ser admitido para receber HV com solução salina a 0,9%, a 1mL/kg/h, 24 horas antes, durante e 12 horas após o procedimento. Entre 48-72 horas após a exposição ao MCI, colheu-se amostra de sangue para análise dos níveis de creatinina sérica (método baseado na reação Jaffe), a fim de avaliar a função renal. Para superar as dificuldades do sistema de saúde pública (má disponibilidade de leitos hospitalares) para internar estes pacientes para um procedimento diagnóstico simples devido ao alto risco de NIC, os diretores do laboratório de cateterização desenvolveram, em 2016, um novo protocolo com HO ambulatorial, evitando assim a hospitalização no dia anterior.

**Figura 1:**
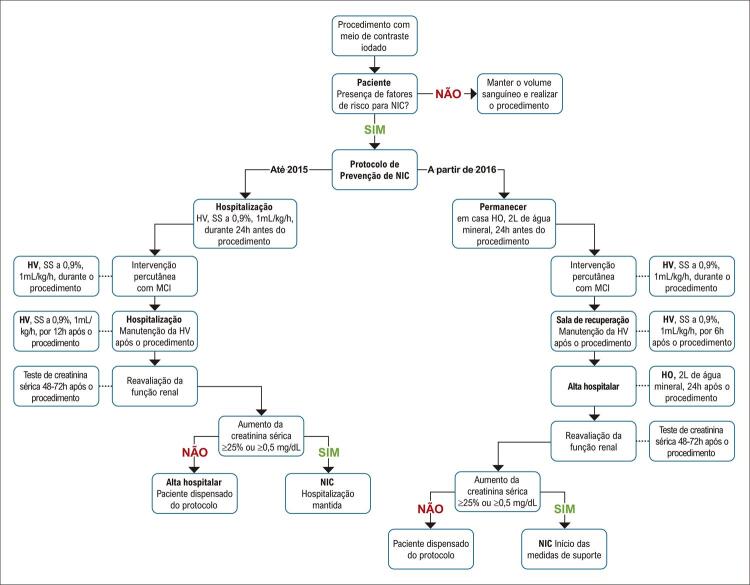
– Desenho do estudo com protocolos para a prevenção de nefropatia induzida por contraste, por via venosa e oral. NIC: Nefropatia induzida por contraste; MCI: Meio de contraste iodado; HV: hidratação venosa; SS: Solução salina; HO: hidratação oral.

Conforme esse novo protocolo, os pacientes eram aconselhados a beber 2 litros de água oralmente, em casa, 24 horas antes e 24 horas após a exposição ao MCI. Durante o tempo de espera e durante o procedimento, realizou-se HV com solução salina a 0,9%, 1mL/kg/h, permanecendo na sala de recuperação após o procedimento por 6 horas, após as quais o paciente recebia alta. Entre 48-72 horas após o procedimento, o paciente retornava ao hospital para recolher amostra de sangue para avaliar o nível de creatinina sérica (método baseado na reação Jaffe). Se não fosse detectado qualquer NIC, o paciente recebia alta definitiva, caso contrário, o paciente era convocado a uma avaliação por cardiologistas e nefrologistas ( Figura 1 ).

Em todos os pacientes, foi administrada uma MCI não iônico e de baixa osmolaridade ou isosmolar, utilizando o menor volume possível.

### Coleta de dados

**Figura 2:**
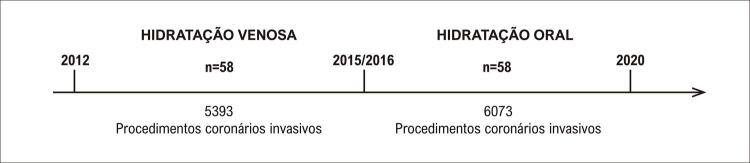
– Linha do tempo com a seleção dos participantes do estudo.

Realizou-se a coleta de dados retrospectivamente através dos prontuários médicos físicos e eletrônicos do Serviço de Arquivo Médico e também através do Portal de Sistemas do Hospital das Clínicas da Universidade Estadual de Campinas - UNICAMP. Analisaram-se 5393 procedimentos de indivíduos submetidos a HV entre janeiro de 2012 e dezembro de 2015 e 6073 procedimentos de indivíduos submetidos a HO entre janeiro de 2016 e janeiro de 2020 ( Figura 2 ).

Coletaram-se os seguintes dados: idade; sexo; raça; peso; tabagismo; níveis de creatinina sérica antes e depois do procedimento; data dos procedimentos; histórico de doenças cardiovasculares como hipertensão, insuficiência cardíaca, infarto do miocárdio e acidente vascular cerebral anterior; histórico de doença renal; histórico de doenças metabólicas como DM e dislipidemia; tipo e volume (mL) de MCI aplicado; procedimento realizado (cateterismo cardíaco e/ou angioplastia coronária) e medicações em uso pelos pacientes. A depuração de creatinina foi calculada usando a equação de Cockcroft-Gault.

Todos os dados foram coletados e verificados por apenas 2 membros da equipe de pesquisa.

### Desfecho

O desfecho primário foi o desenvolvimento de NIC em pacientes submetidos a procedimentos eletivos de cateterismo cardíaco e/ou angioplastia coronária.

### Análise estatística

Para a análise do desfecho primário, os pacientes foram dicotomizados de acordo com a presença ou ausência de NIC e aplicou-se o teste de Fisher. Utilizou-se o teste de Mann-Whitney ou o teste *t* de Student não pareado para comparar dados clínicos e laboratoriais, como idade, creatinina sérica, TFGe e volume de MCI, de acordo com a distribuição dos dados, que foram verificados pelo teste de Shapiro-Wilk. Para todos os outros dados, realizou-se uma dicotomização e aplicou-se o teste de Fisher. Variáveis categóricas foram expressas como porcentagem (%) e número absoluto e variáveis contínuas como média e desvio padrão para dados normalmente distribuídos, ou mediana e intervalo interquartil para dados não distribuídos normalmente. Procedeu-se à análise de regressão logística multivariada para analisar os parâmetros de creatinina sérica basal, volume de MCI, terapia antiplaquetária dupla e heparinização, com o desenvolvimento da NIC como fator dependente. A significância estatística adotada foi α=0,05. Todas as análises estatísticas foram realizadas usando os programas GraphPad Prism, versão 6 para Windows (GraphPad Software, San Diego, CA, USA) e SigmaPlot, versão 12 (Systat Software Inc).

## Resultados

Neste estudo retrospectivo, analisaram-se 11 466 registros e foram coletados dados do período de janeiro de 2012 e janeiro de 2020. Foram selecionados 116 indivíduos que participaram do “Protocolo de Prevenção de NIC” que preencheram os critérios de inclusão e exclusão, onde 58 pacientes receberam HV e 58 pacientes receberam HO ( Figura 2 ).

### Dados demográficos, clínicos e de medicamentos dos sujeitos do estudo

As características gerais basais dos 116 pacientes encontram-se na Tabela 1 . Comparando os grupos, não houve diferenças de idade, sexo, hipertensão, raça, DM, dislipidemia, DRC, insuficiência cardíaca, acidente vascular cerebral e TFGe. Entretanto, constatou-se que aqueles que receberam HV apresentavam percentual maior de infarto do miocárdio prévio, níveis mais altos de creatinina sérica basal, administraram o maior volume de MCI e realizaram mais procedimentos para cateterismo cardíaco mais ICP. A tabela 2 mostra a terapia medicamentosa.

**Tabela 1:** – Dados demográficos e clínicos da população estudada

Características	Hidratação Venosa (n= 58)	Hidratação oral (n= 58)	Valor de p
Idade (anos)	67 ± 10	69 ± 9	0,09
Homens n (%)	42 (72)	34 (59)	0,17
Cor branca n (%)	47 (81)	51 (88)	0,44
Fumante n (%)	27 (47)	33 (57)	0,35
Hipertensão arterial n (%)	55 (95)	56 (97)	1,00
Diabetes *mellitus* n (%)	34 (59)	24 (41)	0,09
Dislipidemia n (%)	45 (78)	45 (78)	1,00
Doença renal crônica n (%)	42 (72)	31 (53)	0,05
Insuficiência cardíaca n (%)	12 (21)	18 (31)	0,29
Infarto do miocárdio anterior n (%)	33 (57)	20 (34)	0,02
Acidente vascular cerebral n (%)	7 (12)	8 (14)	1,00
Creatinina sérica basal (mg/dL)	1,77 (1,29 – 2,16)	1,44 (1,18 – 1,87)	0,03
TFGe basal (mL/min)	39,89 (32,11 – 57,57)	41,88 (35,40 – 49,43)	0,57
Volume de MCI (mL)	100 (50 – 100)	60 (50 – 100)	<0,001
AC n (%)	41 (71)	56 (97)	<0,001
ICP n (%)	2 (3)	0 (0)	0,50
AC + ICP n (%)	15 (26)	2 (3)	0,001

*A idade está representada como média ± DP. Os outros dados são expressos em n (%) ou mediana e intervalo interquartil. TFGe: taxa de filtração glomerular estimada; MCI: meio de contraste iodado AC: angiografia coronariana; ICP: intervenção coronariana percutânea.*

**Tabela 2:** – Medicamentos utilizados pela população estudada

Medicamentos	Hidratação Venosa (n= 58)	Hidratação oral (n= 58)	Valor de p
**Drogas anti-hipertensivas**			
IECAs n (%)	18 (31)	22 (38)	0,56
BRAs n (%)	19 (33)	19 (33)	1,00
Diuréticos n (%)	30 (53)	37 (64)	0,26
BCCs n (%)	19 (33)	21 (36)	0,84
β-bloqueadores n (%)	37 (64)	45 (77)	0,15
Vasodilatadores n (%)	13 (22)	12 (21)	1,00
Simpaticomiméticos n (%)	3 (5)	5 (9)	0,72
**Hipoglicemiantes**			
Biguanidas n (%)	10 (17)	7 (12)	0,60
Sulfonilureias n (%)	3 (5)	2 (3)	1,00
IDPP-4 n (%)	0 (0)	2 (3)	0,49
Insulinas n (%)	22 (38)	12 (21)	0,06
**Hipolipemiantes**			
Estatinas n (%)	40 (69)	42 (72)	0,84
Fibratos n (%)	3 (5)	5 (9)	0,72
Ezetimiba n (%)	0 (0)	3 (5)	0,24
**Outras classes farmacológicas**			
AAS n (%)	42 (72)	41 (71)	1,00
Inibidores dos receptores P2Y12 n (%)	30 (52)	14 (24)	0,004
TAPD n (%)	29 (50)	11 (19)	<0,001
Anticoagulantes orais n (%)	1 (2)	0 (0)	1,00
Heparinização n (%)	21 (36)	0 (0)	<0,001
Anti-inflamatórios n (%)	5 (9)	3 (5)	0,72

*Os valores são expressos em número e percentual. IECAs: inibidores de enzimas conversoras de angiotensina; BRAs: bloqueadores de receptores de angiotensina; BCCs: bloqueadores de canais de cálcio; IDPP-4: inibidores da dipeptidil peptidase-4; AAS: ácido acetilsalicílico; TAPD: terapia antiplaquetária dupla (inibidores P2Y12 + AAS).*

A análise multivariada mostrou que a creatinina sérica basal [RC 1,457; IC95% 0,75 - 2,82; p=0,46], volume de contraste [RC 0,998; IC95% 0,99 - 1,01; p=0,80], terapia antiplaquetária dupla [RC 1,678; IC95% 0,46 - 6,12; p=0,43] e uso de heparina [RC 0,979; IC95% 0,19 - 5,10; p=0,98] não interferiu no desenvolvimento de NIC na população estudada.

Apenas 2 pacientes do grupo HV receberam MCI não iônico isosmolar (iodixanol) e todos os outros pacientes do estudo receberam MCI não iônico de baixa osmolaridade (iobitridol, iopamidol, ioexol ou ioversol) (dados não revelados).

### Incidência de NIC

Observou-se NIC em 9/58 pacientes (15%) no grupo HV e em 7/58 pacientes (12%) no grupo HO (p=0,68; Figura central ). Observou-se aumento ≥0,5mg/dL da creatinina sérica em 6 pacientes (66%) do grupo HV e 3 pacientes (43%) do grupo HO e 4 pacientes em cada grupo (44% e 57%, respectivamente) apresentaram aumento ≥25% após a administração de MCI (dados não revelados).

**Figura Central:**
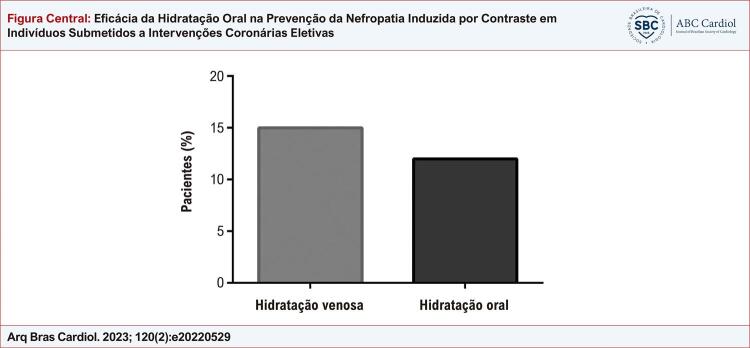
: Eficácia da Hidratação Oral na Prevenção da Nefropatia Induzida por Contraste em Indivíduos Submetidos a Intervenções Coronárias Eletivas

Incidência de nefropatia induzida por contraste em ambos os protocolos de estudo. Os dados são expressos em porcentagem. Teste de Fisher; p=0,68.

## Discussão

Nosso principal resultado é que a HO pode auxiliar no processo de prevenção de NIC antes e depois de procedimentos percutâneos eletivos e pode ser tão eficaz quanto a infusão de solução salina intravenosa a 0,9%. Deve-se enfatizar que todos os pacientes estavam em alto risco de desenvolver NIC, e o grupo selecionado é compatível com outros pacientes submetidos a procedimentos cardíacos eletivos na prática clínica atual.

Os laboratórios de cateterização seguem recomendações específicas a fim de reduzir a incidência de NIC, como o uso de MCI não iônico de baixa osmolaridade e no menor volume possível,^15^ mas ainda assim os casos de NIC ainda ocorrem. Por isso, é essencial associar outras alternativas, como a hidratação. Atualmente, a HV é a mais indicada para a prevenção de NIC,^15 - 18^ porém estudos mais recentes mostram resultados favoráveis à administração de fluidos oralmente em ICP.^22 - 28^ Zhang et al.^19^ conduziram uma metanálise e observaram que a HO foi tão eficaz quanto a HV em pacientes submetidos a angiografia coronariana ou intervenção para prevenção de NIC (5,88% vs. 8,43%; RC: 0,73; IC95%: 0,36-1,47; p>0,05). Esses resultados tiveram um impacto tal que uma diretriz recente do Instituto Nacional de Excelência em Saúde e Cuidados (NICE) do Reino Unido recomenda a HO antes e depois de procedimentos com MCI.^29^

É importante notar que todos esses estudos têm variação metodológica e diferenças nas populações estudadas, resultando em uma variação considerável de 1 a 50% na incidência de NIC com a administração de HO em ICPs invasivos.^20 , 23 - 27 , 30^ Além disso, nenhum estudo comparativo indicou o volume ideal de HO. Em nosso protocolo, o volume de ingestão de água foi padronizado em 2 litros antes e depois dos procedimentos, sem ajuste para peso ou condições clínicas dos pacientes, sendo a maior ingesta de líquidos entre todos os estudos anteriores.^23 - 26^

Neste estudo, os pacientes que receberam HV apresentavam condição clínica mais grave, devido a maior incidência de infarto agudo do miocárdio prévio e níveis mais elevados de creatinina sérica basal quando comparados com os pacientes que receberam HO. Dependendo da complexidade do procedimento, é necessário utilizar diferentes volumes de MCI; assim pacientes graves, como possivelmente os casos do grupo de HV, necessitaram tanto de procedimentos de angiograma quanto de ICP, exigindo volume maior de MCI, causando assim mais NIC. Mesmo nesses pacientes, o volume de MCI administrado foi igual ou menor ao encontrado em outros estudos randomizados que comparavam os protocolos de HO e HV.^20 , 22 - 25 , 27^

O perfil dos pacientes do grupo HV também refletiu o uso de medicamentos, com maior uso de inibidores P2Y12 (clopidogrel), terapia antiplaquetária dupla (ácido acetilsalicílico mais clopidogrel) e heparinização (enoxaparina ou heparina), porém isso não parece ter afetado a incidência de NIC.

Nosso trabalho apresenta várias limitações, inerentes a estudos observacionais. Primeiro, é um estudo observacional retrospectivo, o que impossibilitou a randomização dos pacientes, resultando na heterogeneidade dos grupos. Em segundo lugar, foi realizado em um único centro e com um tamanho de amostra relativamente pequeno, o que confere baixo poder estatístico. Também sugerimos que a extrapolação dos resultados não é recomendada para procedimentos radiológicos utilizando MCI intravenoso. Finalmente, procedimentos coronarianos invasivos podem levar a um processo de embolia de colesterol das artérias renais e, portanto, insuficiência renal aguda após alguns dias, tornando-se um fator de confusão para o MCI.^31^ Essa complicação é pouco relatada e pode ocorrer tanto em pacientes que recebem HV como naqueles que recebem HO. Além disso, nosso estudo foi projetado para comparar a incidência de NIC entre estratégias de HO e HV. Portanto, não foi projetado para avaliar resultados a longo prazo, como mortalidade ou internação hospitalar prolongada.

Nossos resultados corroboram resultados anteriores que sugerem que a HO poderia ser usado na prática clínica, para potencialmente reduzir os custos hospitalares, melhorando o rodízio de leitos hospitalares e propiciar menos internação hospitalar para o paciente. Entretanto, são necessários ensaios clínicos aleatórios e multicêntricos mais cautelosos para confirmar essas descobertas.

## Conclusão

De acordo com os dados analisados, podemos sugerir que um protocolo de HO, em casa, pelo paciente, é tão eficaz quanto o de HV realizado em hospital, visando a proteção renal de indivíduos suscetíveis a desenvolver NIC em procedimentos eletivos de cateterismo cardíaco e angioplastia coronária.
