# Supplementary material for: Eficácia da Hidratação Oral na Prevenção da Nefropatia Induzida por Contraste em Indivíduos Submetidos a Intervenções Coronárias Eletivas
Source: Arq Bras Cardiol. 2023 Feb 13;120(2):e20220529. [Article in Portuguese] doi: 10.36660/abc.20220529 (PMC9972663; doi:10.36660/abc.20220529)
Supplement: Translation

# Translation: Effectiveness of Oral Hydration in Preventing Contrast-Induced Nephropathy in Individuals Undergoing Elective Coronary Interventions

**Authors:** Mariana Rodrigues Pioli, Renata Muller Couto, José de Arimatéia Francisco, Diego Quilles Antoniassi, Célia Regina de Souza, Matheus Ynada de Olivio, Gabriel Forato Anhê, Silvio Giopatto, Andrei C. Sposito, Wilson Nadruz, Otavio Rizzi Coelho-Filho, Rodrigo Modolo

**Affiliations:** 1 Brasil Universidade Estadual de Campinas Faculdade de Ciências Médicas – Departamento de Medicina Translacional, Programa de Farmacologia, Campinas, SP – Brasil; 2 Brasil Universidade Estadual de Campinas Faculdade de Ciências Médicas – Departamento de Medicina Interna – Divisão de Cardiologia, Campinas, SP – Brasil; 3 Brasil Universidade Estadual de Campinas (UNICAMP) – Laboratório de Aterosclerose e Biologia Vascular (Atherolab), Campinas, SP – Brasil; 4 Brasil Universidade Estadual de Campinas (UNICAMP) – Disciplina de Cardiologia, Campinas, SP – Brasil

**Keywords:** Acute Kidney Injury, Contrast Media, Angioplasty, Cardiac Catheterization

## Abstract

**Background:**

Contrast-induced nephropathy (CIN) is defined as worsening renal function, represented by an increase in serum creatinine of ≥ 25% or ≥ 0.5 mg/dL up to 72 h after exposure to iodinated contrast medium (ICM). The most effective preventive measure to date is intravenous hydration (IVH). Little is known about the effectiveness of outpatient oral hydration (OH).

**Objetive:**

To investigate whether outpatient OH with water is as effective as IVH with 0.9% saline solution in preventing CIN in elective coronary procedures.

**Methods:**

In this retrospective observational study, we analyzed the medical records and laboratory data of individuals undergoing percutaneous coronary procedures with ICM. Data collected between 2012 and 2015 refer to individuals who underwent IVH and those collected between 2016 and 2020 (after implementation of an OH protocol) correspond to individuals who underwent OH at home before and after coronary procedures as instructed by the nursing team. Statistical significance was established at α = 0.05.

**Results:**

In total, 116 patients were included in this study: 58 in the IVH group and 58 in the OH group. An incidence of CIN of 15% (9/58) was observed in the group that received IVH and an incidence of 12% (7/58) was seen in the group that received OH (p = 0.68).

**Conclusion:**

The OH protocol, performed by the patient, appears to be as effective as the in-hospital IVH protocol for the renal protection of individuals susceptible to CIN in elective coronary interventions. These findings should be put to test in larger trials.

**Central Illustration:** : Effectiveness of Oral Hydration in Preventing Contrast-Induced Nephropathy in Individuals Undergoing Elective Coronary Interventions

Incidence of contrast-induced nephropathy in both studied protocols. Data expressed as percentages. Fisher’s test; p = 0.68. IV: intravenous.

## Introduction

Contrast-induced nephropathy (CIN) was described in 1954 by Bartels et al.^1^and defined by Mehran et al.^2^as an increase in serum creatinine of ≥ 25% or ≥ 0.5 mg/dL up to 72 h after exposure to iodinated contrast medium (ICM). It is considered a highly incident iatrogenesis in coronary interventions, reaching up to 2% of the population exposed to ICM^3 , 4^or up to 50% of high-risk populations.^5 , 6^Moreover, it is strongly associated with unfavorable clinical outcomes such as long-term morbidity and mortality.^7 , 8^

The development in CIN is associated with both ICM characteristics^9 , 10^and the patient’s clinical condition, since individuals with preexisting renal failure, diabetes, and older adults are more prone to this outcome.^11 , 12^Prophylactic measures have been reported to be effective in reducing the incidence of CIN, namely: identification of risk factors, use of the lowest possible volume of ICM, and renal protection before and after the procedure by means of intravenous hydration (IVH) with 0.9% saline solution.^13 , 14^

Despite being safe and recommended by several guidelines,^15 - 18^IVH has characteristics that sometimes limit its application, such as increased hospitalization time, which generates high costs for the hospital and discomfort for the patients. An alternative strategy such as oral hydration (OH) could be a relevant option, since in addition to causing an adequate volume expansion, it is easily performed before and after the procedure, low-cost, and comfortable for the patient.

Previous studies have shown that OH may reduce the incidence of CIN,^19^but others showed superiority of IVH.^20^In view of these conflicting results, we aimed to investigate whether OH with water, pre- and post-ICM administration, is as effective as IVH with 0.9% saline solution in the renal protection of individuals susceptible to CIN in elective procedures for cardiac catheterization and coronary interventions.

## Methods

This study was approved by the Research Ethics Committee of the School of Medical Sciences of UNICAMP (#4.124.863 and CAAE: 33427720 2 0000 5404). Since this is a retrospective observational study, there was no need for an informed consent form by the research participants, as approved by the ethics committee.

### Patient population and definition of CIN

This study selected 116 consecutive patients who underwent elective procedures of cardiac catheterization and/or percutaneous coronary intervention (PCI) between January 2012 and January 2020 and were at high risk for developing CIN (criteria described below). This is a single-center study from the Cardiac Catheterization Laboratory of Hospital de Clínicas da Universidade Estadual de Campinas – UNICAMP.

All patients underwent medical history evaluation with the nursing team for assessing the risk of developing CIN, which was defined as an increase in serum creatinine of ≥ 25% or ≥ 0.5 mg/dL up to 72 h after exposure to ICM.^2^Patients with serum creatinine > 1.5 mg/dL or an estimated glomerular filtration rate (eGFR) between 40 and 60 mL/min were automatically included in the “CIN Prevention Protocol.”

For those with normal serum creatinine levels, other clinical features (related to worsening renal function) were assessed to classify them as at risk for developing CIN. The factors considered for this risk assessment were: older adults (> 75 years old); preexisting comorbidities such as diabetes mellitus (DM), hypertension, and chronic kidney disease (CKD); hemodynamic instability; and use of nephrotoxic drugs.^12 , 21^Exclusion criteria were as follows: dialysis patients, urgency and emergency cases, individuals with congestive heart failure (functional class III and IV), and those without information in their medical records.

### Study protocol

Until 2015, patients included in the “CIN Prevention Protocol” should be admitted to receive IVH with 0.9% saline solution at 1 mL/kg/h 24 h before, during, and 12 h after the procedure. Between 48 and 72 h after exposure to ICM, a blood sample was collected for analyzing serum creatinine levels (method based on the Jaffe reaction) to assess renal function. In view of the difficulties of the public health system (poor availability of hospital beds) to hospitalize these patients for a simple diagnostic procedure due to the high risk of CIN, the directors of the catheterization laboratory developed a new protocol with outpatient OH in 2016, thus precluding hospitalization a day before the procedure.

In this new protocol, patients were advised to drink 2 liters of water at home, 24 h before and 24 h after exposure to ICM. During the waiting time and throughout the procedure, IVH with 0.9% saline solution at 1 mL/kg/h was performed, continuing after the procedure (in the recovery room) for 6 h, after which the patient was discharged. Between 48 and 72 h after the procedure, the patient returned to the hospital for blood sample collection to assess serum creatinine levels (method based on the Jaffe reaction). If no CIN was detected, the patient was discharged definitively; otherwise, the patient was referred to an evaluation by cardiologists and nephrologists ( Figure 1 ).

**Figure 1:** – Study design with protocols for the prevention of contrast-induced nephropathy through intravenous and oral hydration. CIN: contrast-induced nephropathy; ICM: iodinated contrast medium; IV: intravenous; SS: saline solution.

The lowest possible volume of a non-ionic and low-osmolar or iso-osmolar ICM was administered to all patients.

### Data collection

Data collection was performed retrospectively from the physical and electronic medical records of the Medical Archive Service and also through the Systems Portal of Hospital de Clínicas da Universidade Estadual de Campinas – UNICAMP. Between January 2012 and December 2015, we analyzed 5,393 procedures in individuals who underwent IVH and, between January 2016 and January 2020, 6,073 procedures in individuals who underwent OH ( Figure 2 ).

**Figure 2:** – Timeline of study participant selection.

The following data were collected: age; sex; race; weight; smoking status; serum creatinine levels before and after the procedure; date of procedures; history of cardiovascular diseases such as hypertension, heart failure, previous myocardial infarction, and stroke; history of kidney disease; history of metabolic diseases such as DM and dyslipidemia; type and volume (mL) of ICM used; procedure performed (cardiac catheterization and/or coronary angioplasty); and medications used by the patients. Creatinine clearance was calculated using the Cockcroft-Gault equation.

All data were collected and checked by 2 members of the research team.

### Outcome

The primary outcome was the development of CIN in patients undergoing elective procedures of cardiac catheterization and/or coronary angioplasty.

### Statistical analysis

For analyzing the primary outcome, patients were dichotomized according to the presence or absence of CIN, and the Fisher’s test was applied. For comparing clinical and laboratory data such as age, serum creatinine, eGFR, and ICM volume, the Mann-Whitney test or the unpaired Student’s t-test was applied according to the data distribution, which was verified by the Shapiro-Wilk test. All other data were dichotomized and analyzed by the Fisher’s test. Categorical variables were expressed as percentages (%) and absolute numbers, whereas continuous variables were expressed as means and standard deviations for normally distributed data or median values and interquartile ranges for non-normally distributed data. A multivariate logistic regression analysis was performed to analyze baseline serum creatinine, ICM volume, dual antiplatelet therapy, and heparinization, with the development of CIN as a dependent factor. Statistical significance was established at α = 0.05. All statistical analyzes were performed using GraphPad Prism version 6 for Windows (GraphPad Software, San Diego, CA, USA) and SigmaPlot version 12 (Systat Software Inc., Chicago, IL, USA).

## Results

In this retrospective study, we analyzed 11,466 records and collected data from January 2012 to January 2020. One hundred and sixteen individuals who participated in the “CIN Prevention Protocol” were selected after applying the inclusion and exclusion criteria; 58 patients received IVH and 58 patients received OH ( Figure 2 ).

### Demographic, clinical, and medication use data of the study participants

General baseline characteristics of the 116 patients are shown in Table 1 . When comparing the IVH and OH groups, there were no differences in age, sex, race, hypertension, DM, dyslipidemia, CKD, heart failure, stroke, and eGFR. However, those who received IVH had a higher percentage of previous myocardial infarction, higher baseline levels of serum creatinine, were administered the largest volumes of ICM, and performed a greater number of procedures for cardiac catheterization plus PCI. Table 2 shows the medication therapies taken by the participants.

**Table 1:** – Demographic and clinic data of the study population

Characteristics	IV hydration (n= 58)	Oral hydration (n= 58)	p value
Age (years)	67 ± 10	69 ± 9	0.09
Male n (%)	42 (72)	34 (59)	0.17
White race n (%)	47 (81)	51 (88)	0.44
Smoker n (%)	27 (47)	33 (57)	0.35
Arterial hypertension n (%)	55 (95)	56 (97)	1.00
Diabetes mellitus n (%)	34 (59)	24 (41)	0.09
Dyslipidemia n (%)	45 (78)	45 (78)	1.00
Chronic kidney disease n (%)	42 (72)	31 (53)	0.05
Heart failure n (%)	12 (21)	18 (31)	0.29
Previous myocardial infarction n (%)	33 (57)	20 (34)	0.02
Stroke n (%)	7 (12)	8 (14)	1.00
Serum creatinine, baseline (mg/dL)	1.77 (1.29 – 2.16)	1.44 (1.18 – 1.87)	0.03
eGFR baseline (mL/min)	39.89 (32.11 – 57.57)	41.88 (35.40 – 49.43)	0.57
ICM volume (mL)	100 (50 – 100)	60 (50 – 100)	<0.001
CA n (%)	41 (71)	56 (97)	<0.001
PCI n (%)	2 (3)	0 (0)	0.50
CA + PCI n (%)	15 (26)	2 (3)	0.001

*Age is presented as mean ± standard deviation (SD). All other data are expressed in n (%) or median values and interquartile ranges. IV: intravenous; eGFR: estimated glomerular filtration rate; ICM: iodinated contrast medium; CA: coronary angiogram; PCI: percutaneous coronary intervention.*

**Table 2:** – Medications used by the study population

Characteristics	IV hydration (n= 58)	Oral hydration (n= 58)	p value
**Antihypertensive drugs**			
ACEIs n (%)	18 (31)	22 (38)	0.56
ARBs n (%)	19 (33)	19 (33)	1.00
Diuretics n (%)	30 (53)	37 (64)	0.26
CCBs n (%)	19 (33)	21 (36)	0.84
β-blockers n (%)	37 (64)	45 (77)	0.15
Vasodilators n (%)	13 (22)	12 (21)	1.00
Sympathomimetics n (%)	3 (5)	5 (9)	0.72
**Antidiabetic drugs**			
Biguanide n (%)	10 (17)	7 (12)	0.60
Sulphonylureas n (%)	3 (5)	2 (3)	1.00
DPP-4 inhibitors n (%)	0 (0)	2 (3)	0.49
Insulin n (%)	22 (38)	12 (21)	0.06
**Hypolipidemic drugs**			
Statins n (%)	40 (69)	42 (72)	0.84
Fibrates n (%)	3 (5)	5 (9)	0.72
Ezetimibe n (%)	0 (0)	3 (5)	0.24
**Other pharmacological classes**			
ASA n (%)	42 (72)	41 (71)	1.00
P2Y12 inhibitors n (%)	30 (52)	14 (24)	0.004
DAPT n (%)	29 (50)	11 (19)	<0.001
Oral anticoagulation n (%)	1 (2)	0 (0)	1.00
Heparinization n (%)	21 (36)	0 (0)	<0.001
bn Anti-inflammatory drugs n (%)	5 (9)	3 (5)	0.72

*Values are expressed in numbers and percentages. IV: intravenous; ACEIs: angiotensin-converting enzyme inhibitors; ARBs: angiotensin receptor blockers; CCBs: calcium channel blockers; DPP-4 inhibitors: dipeptidyl peptidase-4 inhibitors; ASA: acetylsalicylic acid; DAPT: dual antiplatelet therapy (P2Y12 inhibitors + ASA).*

A multivariate analysis showed that baseline serum creatinine (odds ratio [OR] 1.457; 95% confidence interval [CI] 0.75-2.82; p = 0.46), contrast volume (OR 0.998; 95% CI 0.99-1.01; p = 0.80), dual antiplatelet therapy (OR 1.678; 95% CI 0.46-6.12; p = 0.43), and use of heparin (OR 0.979; 95% CI 0.19-5.10; p = 0.98) did not interfere with the development of CIN in the studied population.

Only two patients in the IVH group received non-ionic iso-osmolar ICM (iodixanol); all the other patients in this study received nonionic low-osmolar ICM (iobitridol, iopamidol, iohexol, or ioversol) (data not shown).

### Incidence of CIN

CIN was seen in 9/58 patients (15%) in the IVH group and in 7/58 patients (12%) in the OH group (p = 0.68; Central Figure ). Six patients (66%) in the IVH group and 3 patients (43%) in the OH group had increases in serum creatinine levels ≥ 0.5mg/dL, and 4 patients from each group (44% and 57%, respectively) had increases in serum creatinine levels ≥ 25% after ICM administration (data not shown).

## Discussion

Our main result is that OH before and after elective percutaneous procedures may assist in the process of preventing CIN and may be as effective as IV infusion with 0.9% saline solution. It should be emphasized that all patients were at high risk for developing CIN, and the selected group is compatible with other patients undergoing elective cardiac procedures in current clinical practice.

Catheterization laboratories follow specific recommendations in order to reduce the incidence of CIN, such as the use of non-ionic, low-osmolar ICM and the lowest possible contrast volume,^15^but CIN events still manage to occur; it is thus essential to associate these measures with other alternatives such as hydration. Currently, IVH is the most indicated measure for preventing CIN,^15 - 18^although more recent studies show favorable results of the oral administration of fluids in patients undergoing PCI.^22 - 28^A meta-analysis by Zhang et al.^19^analyzed such studies and noticed that OH was as effective as IVH in patients undergoing coronary angiography or intervention for preventing CIN (5.88 vs 8.43%; OR 0.73; 95% CI 0.36-1.47; *p* > 0.05). These results had such an impact that a recent guideline by the National Institute for Health and Care Excellence (NICE) encourages OH before and after procedures using ICM.^29^

Of note, all these studies have methodological variations and differences between studied populations, resulting in considerable variation (ranging from 1 to 50% in the incidence of CIN with OH in percutaneous invasive coronary procedures).^20 , 23 - 27 , 30^In addition, no comparative studies stated the ideal volume for OH. In our protocol, the volume of water ingestion was standardized at 2 liters before and after the procedures, without adjusting for the weight or clinical conditions of patients; this is the largest fluid intake among all studies to date.^23 - 26^

In this study, patients who received IVH had more severe clinical conditions due to a higher incidence of previous acute myocardial infarction and higher baseline serum creatinine levels when compared to patients who received OH. Depending on the complexity of the procedure, different volumes of ICM are required; severely ill patients (as were potentially the cases in the IVH group) thus needed both angiogram and PCI procedures, requiring larger volumes of ICM that could lead to more CIN. Even in these patients, the volume of ICM administered was equal to or less than that found in other randomized studies comparing OH and IVH protocols.^20 , 22 - 25 , 27^

The profile of patients in the IVH group also reflected their medication therapy—with greater use of P2Y12 inhibitors (clopidogrel), dual antiplatelet therapy (acetylsalicylic acid plus clopidogrel), and heparinization (enoxaparin or heparin)—; however, this does not seem to have impacted the incidence of CIN.

Our work has several limitations that are inherent to observational studies. First, it is a retrospective observational study, which made it impossible to randomize patients and resulted in heterogeneous groups. Second, it was carried out in a single center with a relatively small sample size, which gives it low statistical power. We also do not recommend extrapolating the results for radiological procedures using intravenous ICM. Finally, invasive coronary procedures can lead to a process of atheroembolism of the renal arteries and consequent acute renal failure after a few days, becoming a confounding factor for CIN.^31^This complication is underreported and can occur both in patients who receive IVH and those who receive OH. In addition, our study was designed to compare the incidence of CIN between oral and intravenous hydration strategies; therefore, it was not designed to evaluate long-term results such as mortality or prolonged hospital stay.

Our results corroborate previous findings that suggest that OH could be used in clinical practice to potentially reduce hospital costs (improving hospital bed rotation) and provide shorter in-hospital stays for the patients. However, more thorough randomized and multi-center clinical trials are needed to confirm these findings.

## Conclusion

According to the analyzed data, we can suggest that an OH protocol performed at home, by the patient, is as effective as hospital IVH for the renal protection of individuals susceptible to the development of CIN in elective procedures of cardiac catheterization and coronary angioplasty.
